# Supplementary figures and images for: Contrasting determinants for the introduction and establishment success of exotic birds in Taiwan using decision trees models
Source: PeerJ. 2017 Mar 14;5:e3092. doi: 10.7717/peerj.3092 (PMC5354111; doi:10.7717/peerj.3092)

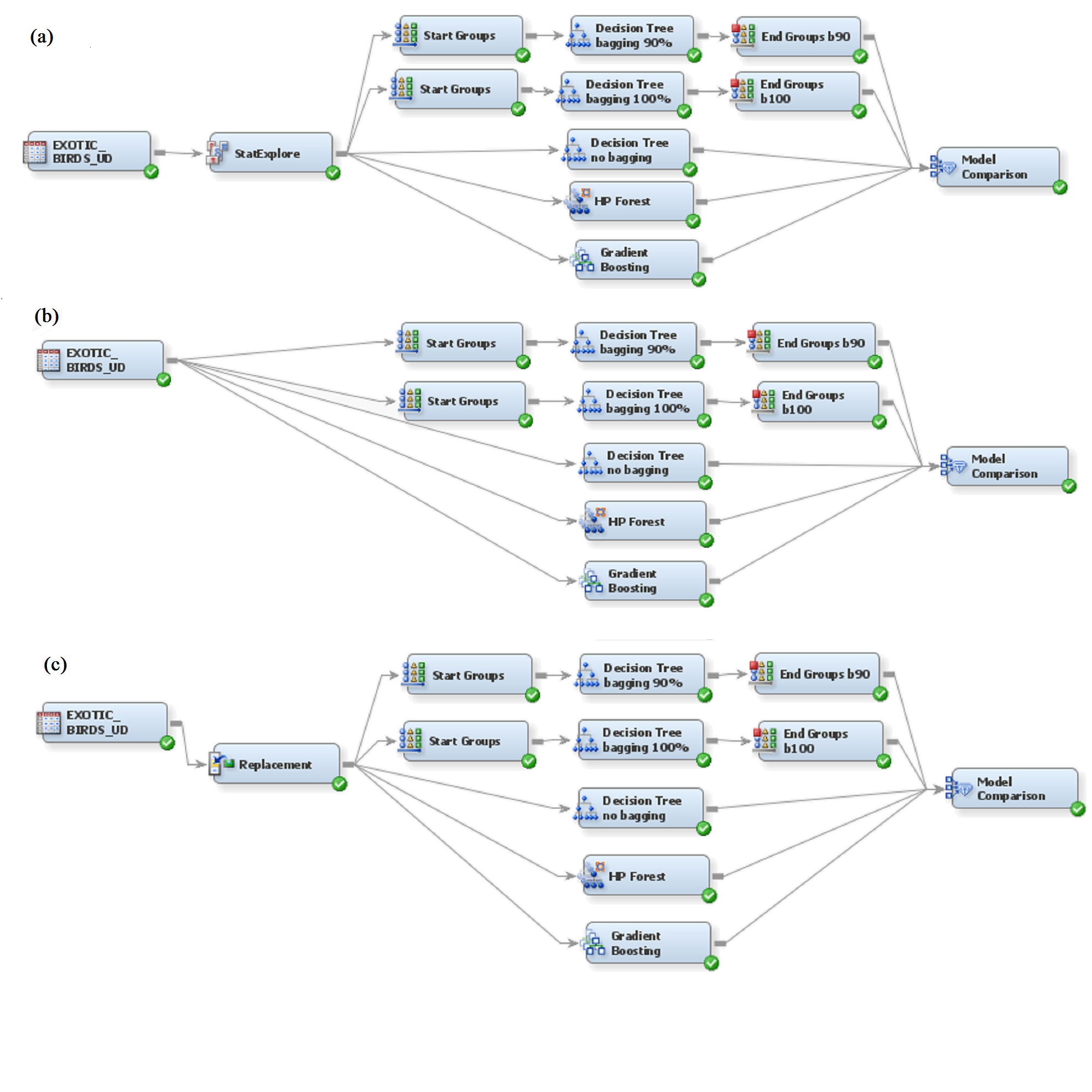

Supplement: Figure S1 [file peerj-05-3092-s002.png]

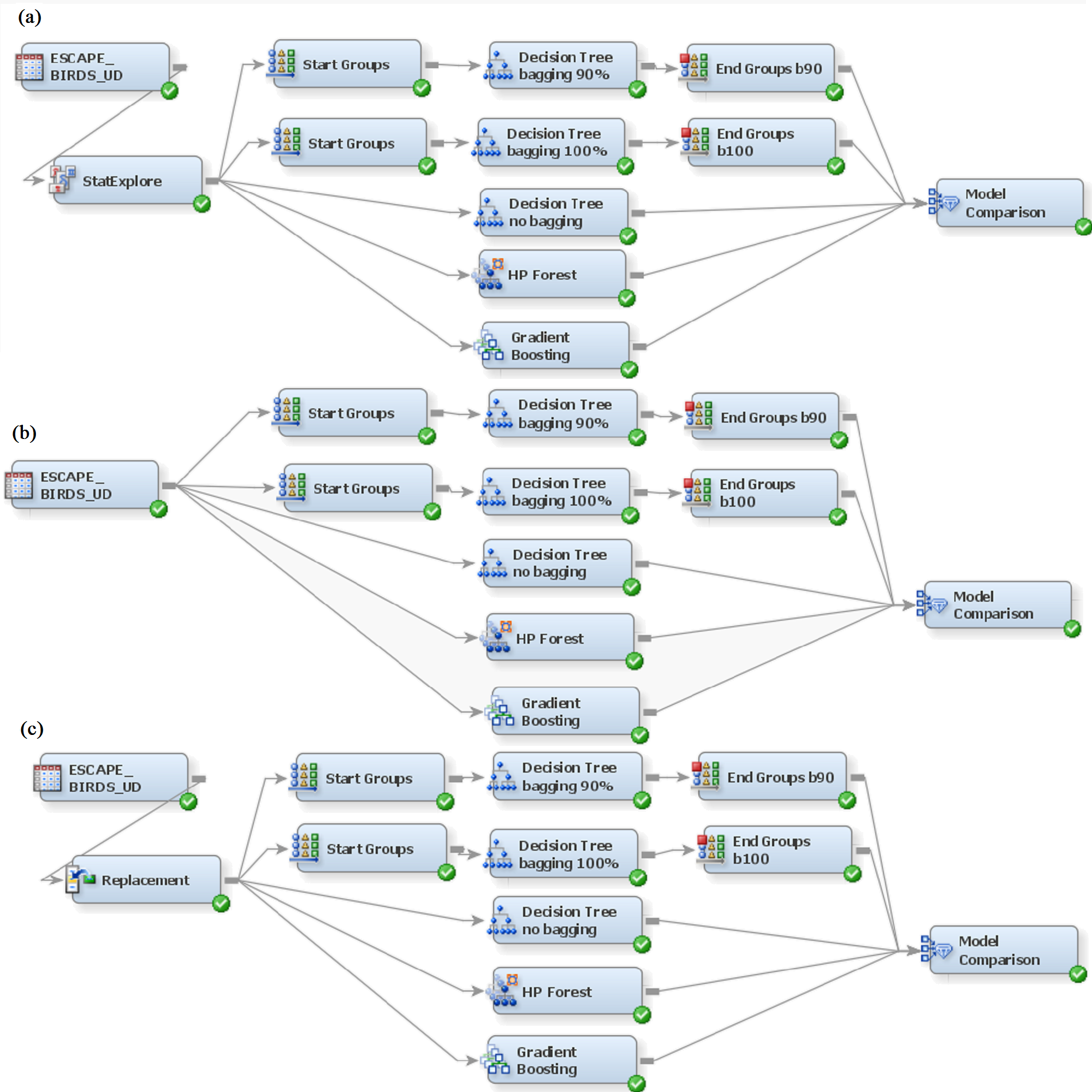

Supplement: Figure S2 [file peerj-05-3092-s003.png]
