# Supplementary material for: Contrasting determinants for the introduction and establishment success of exotic birds in Taiwan using decision trees models
Source: PeerJ. 2017 Mar 14;5:e3092. doi: 10.7717/peerj.3092 (PMC5354111; doi:10.7717/peerj.3092)
Supplement: Supplemental Information 2 — Figure S3. Receiver operating characteristic (ROC) curves of five introduction models: (A) variable treatment I, (B) variable treatment II, and (C) variable treatment III. Figure S4. Classification charts of five introduction models: (A) variable treatment I, (B) variable treatment II, and (C) variable treatment III. Figure S5. Receiver operating characteristic (ROC) curves of five establishment models: (A) variable treatment I, (B) variable treatment II, and (C) variable treatment III. Figure S6. Classifications charts of five establishment models: (A) variable treatment I, (B) variable treatment II, and (C) variable treatment III. [file peerj-05-3092-s004.pdf]

(a)

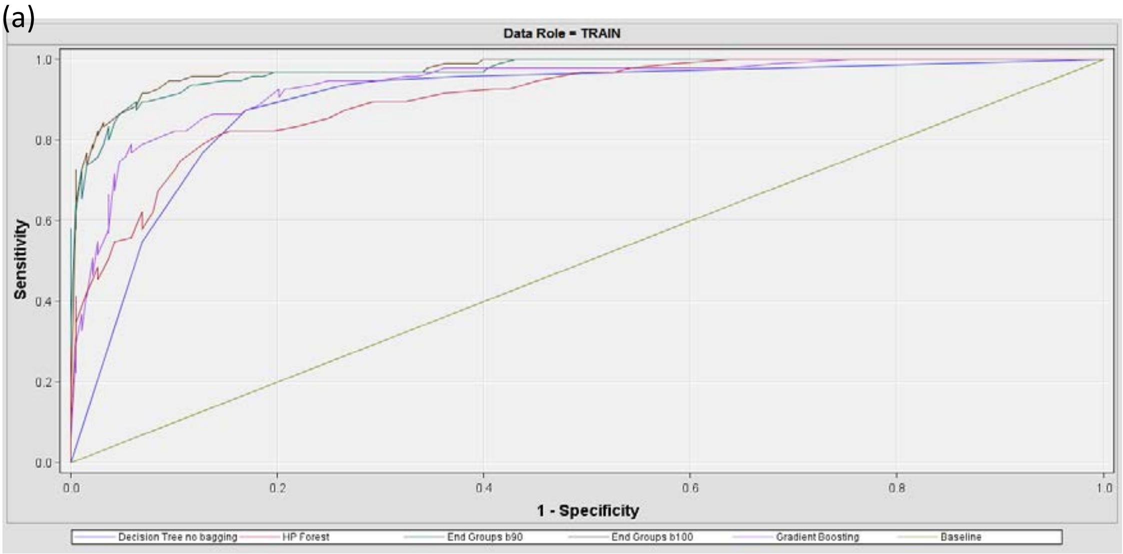

(b)

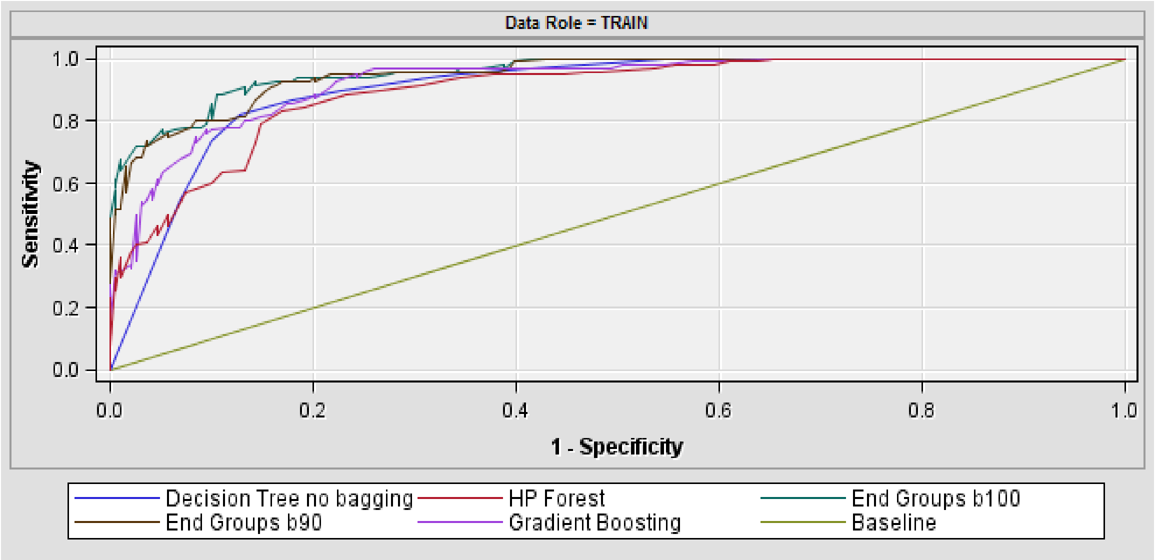

(c)

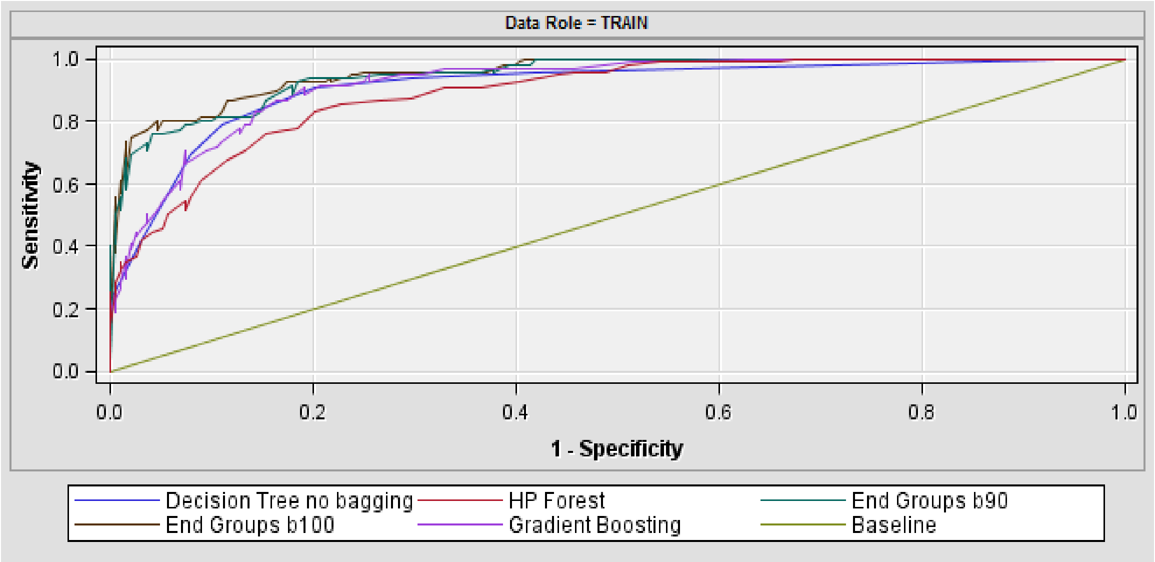

Figure S3. Receiver operating characteristic (ROC) curves of five introduction models: (a) variable treatment I, (b) variable treatment II, and (c) variable treatment III.

(a)

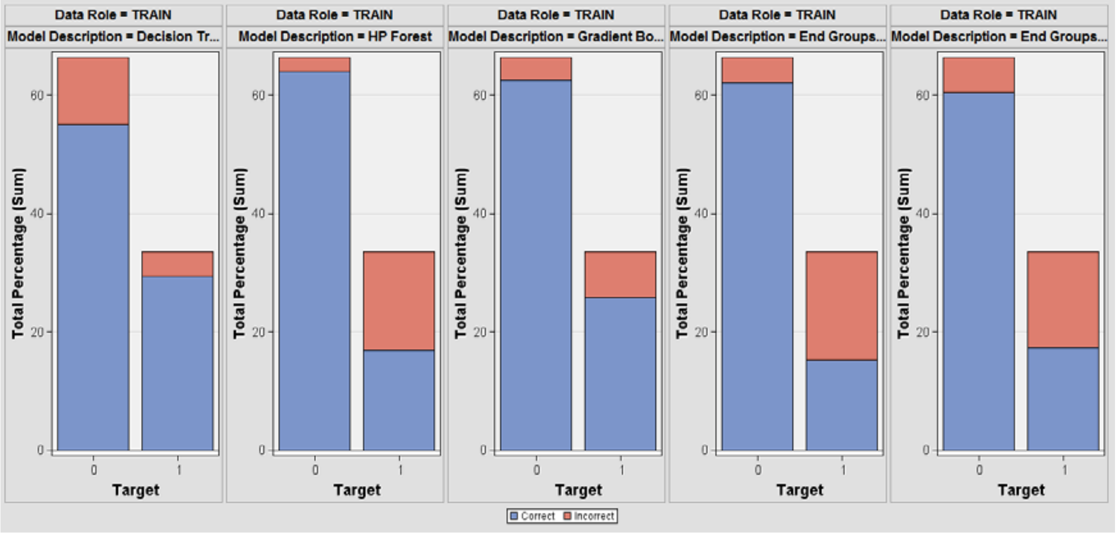

(b)

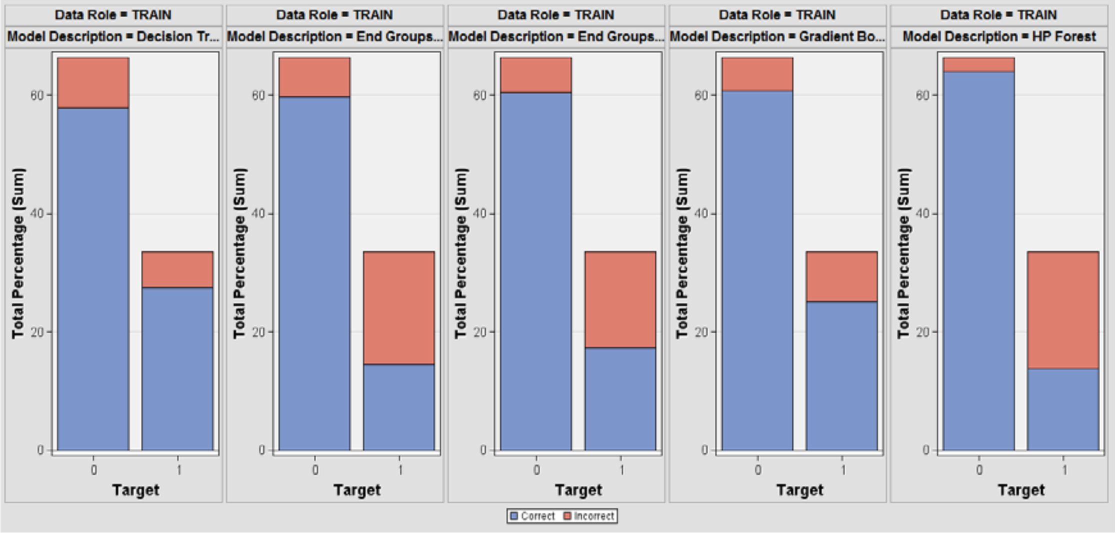

(c)

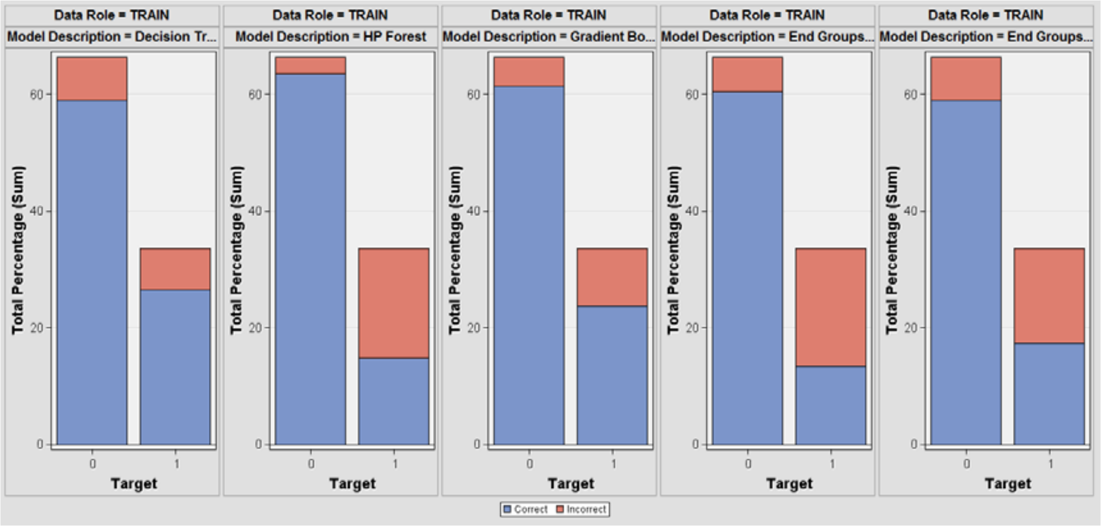

Figure S4. Classification charts of five introduction models: (a) variable treatment I, (b) variable treatment II, and (c) variable treatment III.

(a)

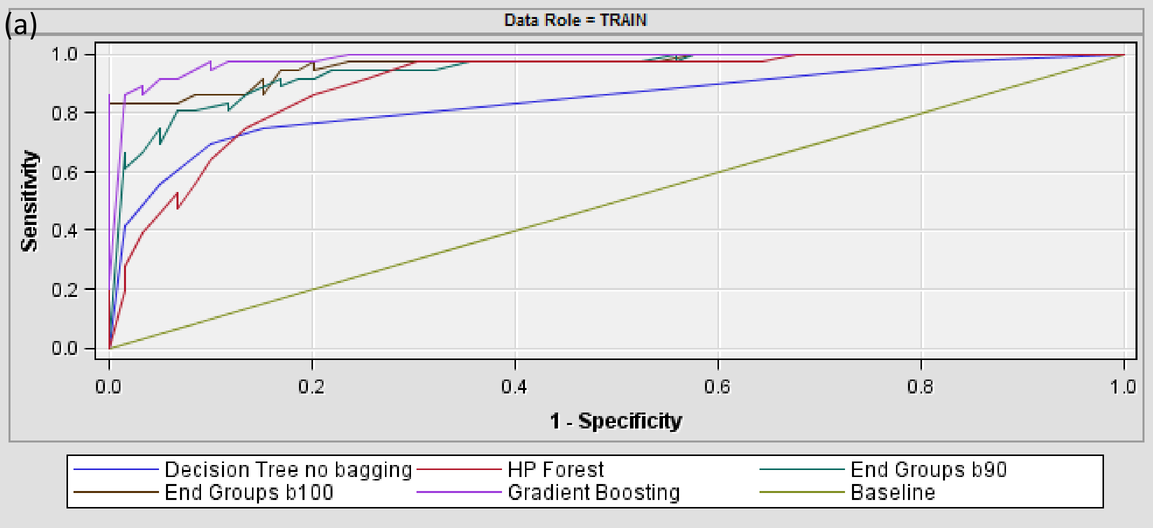

(b)

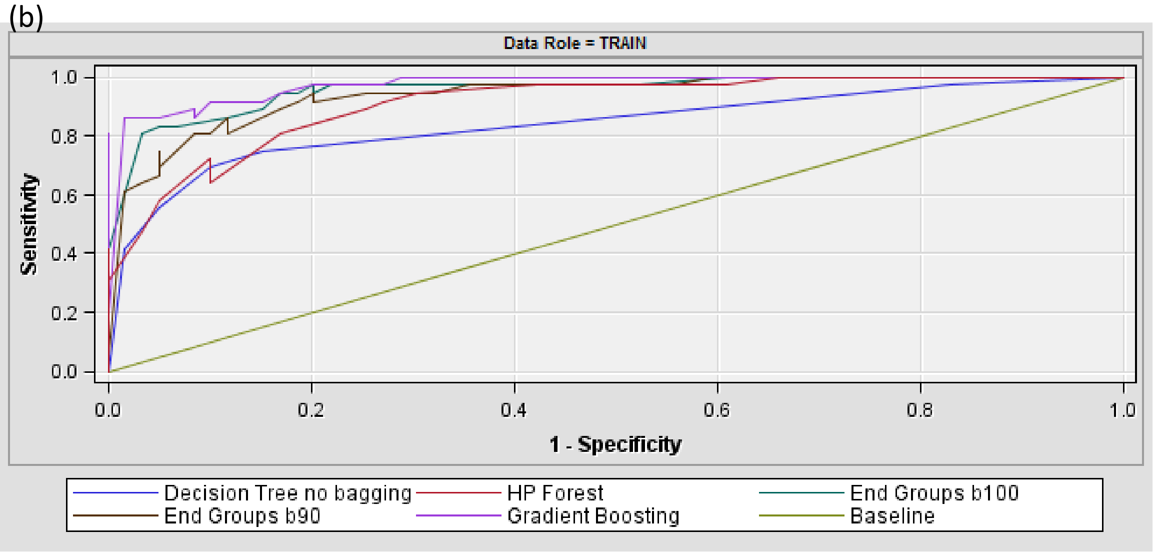

(c)

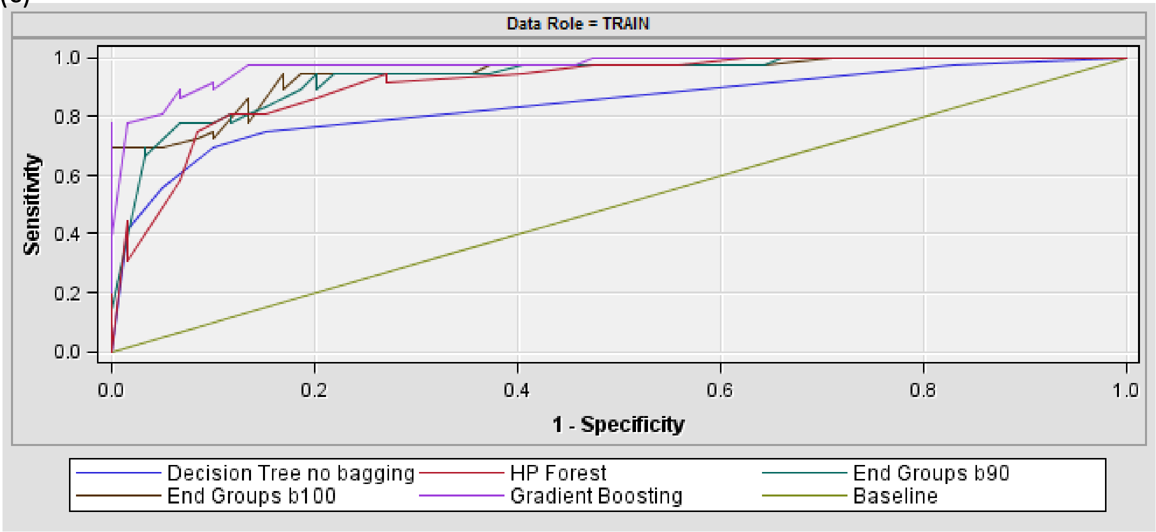

Figure S5. Receiver operating characteristic (ROC) curves of five establishment models: (a) variable treatment I, (b) variable treatment II, and (c) variable treatment III.

(a)

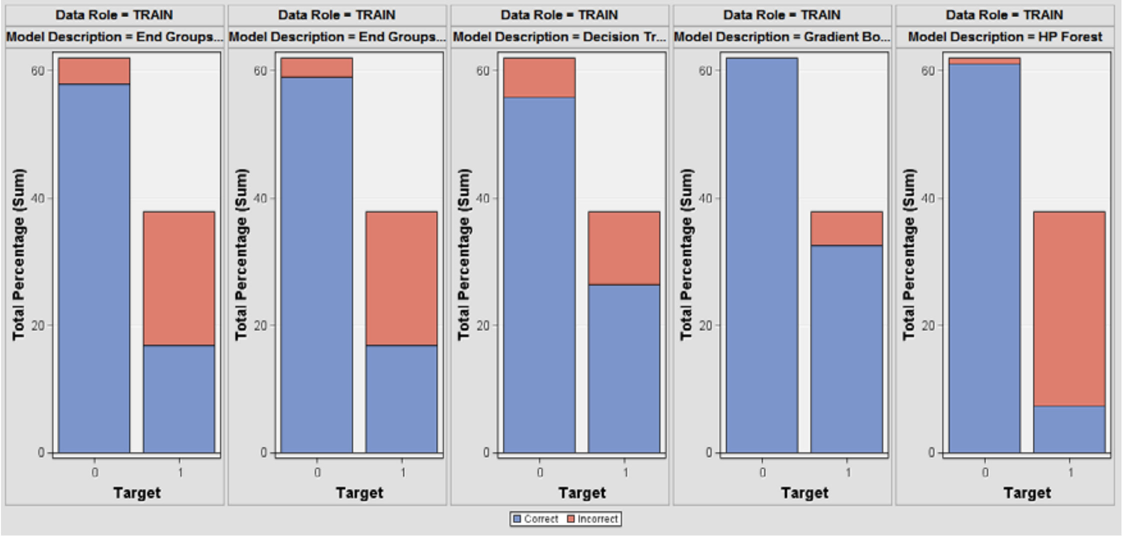

(b)

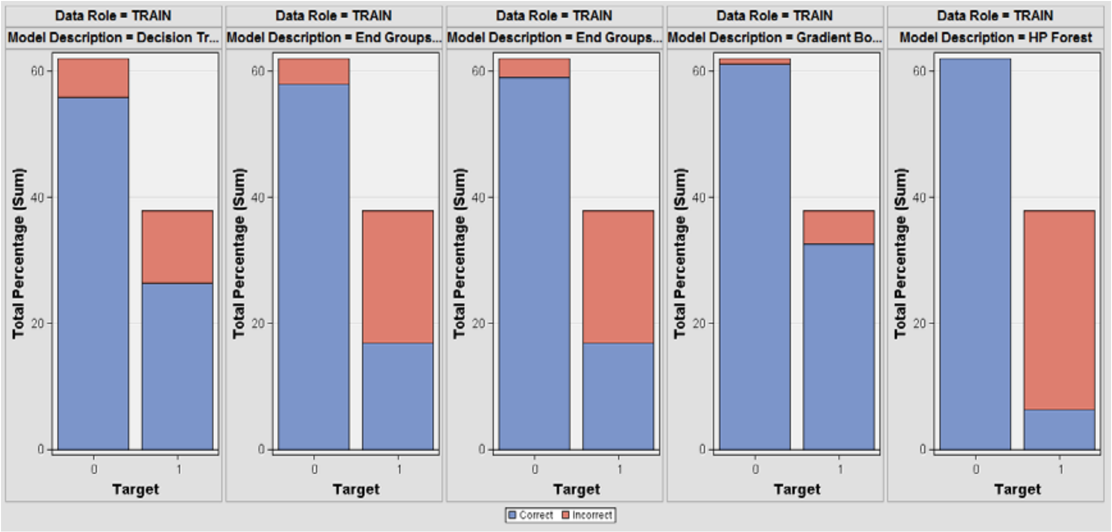

(c)

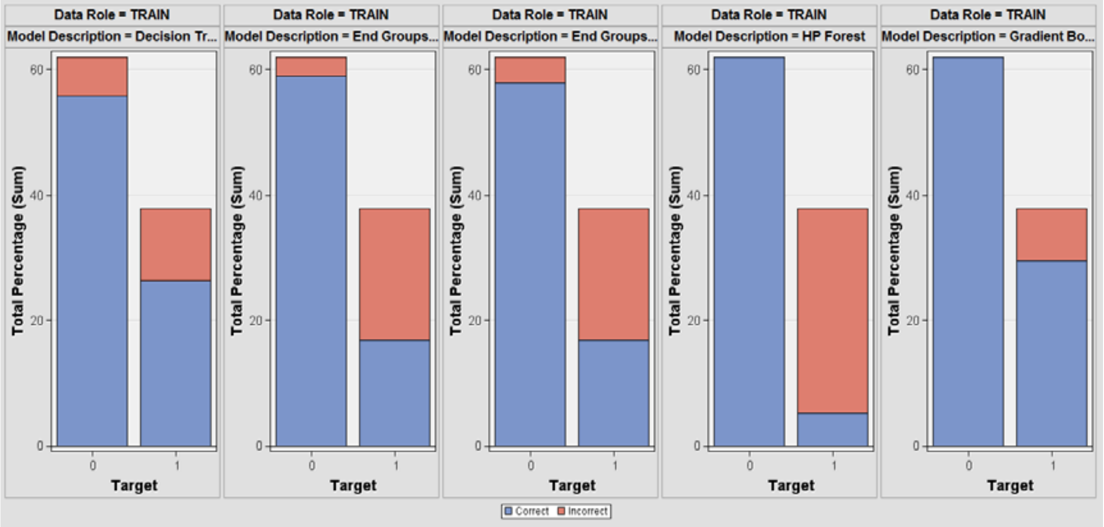

Figure S6. Classification charts of five establishment models: (a) variable treatment I, (b) variable treatment II, and (c) variable treatment III.
